# Dr. William B. Atkinson

**Published:** 1910-01

**Authors:** 


Dr. William B. Atkinson, of Philadelphia, died at his home November 23, 1909, aged 77 years. He served thirty-five years as secretary of the American Medical Association, and his familiar face and voice will be remembered by those who attended the meetings during the period from 1864 to 1899.

